# The *RALF1–eIF4E1* Signaling Axis Mediates Root Hair Elongation, Flowering Time, and Stress Tolerance During Seed Germination and Early Root Growth in *Arabidopsis thaliana*

**DOI:** 10.3390/plants15091369

**Published:** 2026-04-30

**Authors:** Feirong Zeng, Pian Yang, Guixiang He, Aoxue Wang, Yan Gao, Jihong Zhang

**Affiliations:** 1 School of Life and Health Science, Hunan University of Science and Technology, Xiangtan 411201, China; 2 Hunan Engineering Research Center of Lotus Deep Processing and Nutritional Health Sciences, Hunan University of Science and Technology, Xiangtan 411201, China

**Keywords:** *Arabidopsis thaliana*, *RALF1*, *eIF4E1*, root hair, abiotic stress, RALF1-FERONIA-eIF4E1 signaling

## Abstract

To investigate the coordinated role of *RALF1* and *eIF4E1* within the FER signaling module in regulating root hair elongation and stress responses in *Arabidopsis thaliana*, we constructed a *ralf1*/*eif4e1* double mutant via conventional hybridization. Although the roles of the *RALF1* and *eIF4E1* genes are well known, the simultaneous absence of them remains poorly characterized. The double mutant exhibited significantly reduced root hair numbers and elongation and heightened sensitivity to ABA, Cd^2+^, and NaCl stress. The *ralf1*/*eif4e1* double mutant exhibited delayed flowering time and higher numbers of rosette leaves. Fluorescence quantitative PCR analyses revealed that several key genes involved in regulating flowering such as *FT*, *LFY* and *SOC* reached maximum levels in wild-type plants. However, other genes that regulated floral meristem exhibited higher expression levels in the *ralf1* mutant, followed by in wild-type plants. This work provides new insight into the RALF1-FERONIA-eIF4E1 module, demonstrating that it converges environmental cues to coordinately regulate root hair elongation, stress responses, and flowering time in Arabidopsis.

## 1. Introduction

The development of root hairs is an important adaptive feature that enhances plant water absorption and nutrient uptake and improves tolerance to adverse environments. In *Arabidopsis thaliana*, root hair development is tightly regulated by peptide-receptor signaling pathways. Receptor-like kinases (RLKs) constitute one of the largest single-transmembrane receptor families in plants, capable of sensing diverse extracellular signals at the plasma membrane [1]. Among them, FERONIA (FER) is a well-characterized RLK belonging to the Caharanthus roseus RLK1-like (CrRLK1L) subfamily, whose extracellular domain contains malectin-like domains implicated in cell wall integrity sensing [2].

Recent studies have established that FER plays pleiotropic roles in stress responses (e.g., immune responses) and cell growth. For instance, Guo et al. (2018) demonstrated that FER negatively regulates jasmonic acid (JA) signaling to suppress JA-mediated defense responses, thereby balancing growth and immunity [3]. Chen et al. (2020) further reviewed the fact that the FER cytoplasmic domain serves as a signaling node that integrates multiple outputs including ROS production, calcium signaling, and hormone crosstalk [4]. Zhang et al. (2020) summarized that the RALF-FER signaling module links immune responses with cell growth regulation [5].

The rapid alkalinization factor (RALF) peptides are small secreted signals. Upon binding to FER, RALF1 triggers downstream phosphorylation cascades that integrate hormonal cues such as auxin and abscisic acid (ABA) [6,7,8]. Specifically, Du et al. (2016) showed that the RALF1-FER complex inhibits root growth by suppressing plasma membrane H^+^-ATPase activity, leading to apoplastic alkalinization [6]. Chen et al. (2016) revealed that FER interacts with ABI2-type phosphatases to facilitate crosstalk between ABA and RALF signaling, modulating stress responses [7]. Liao et al. (2017) further highlighted that FER acts at the crossroads of hormone signaling (auxin, ABA, ethylene) and stress adaptation [8].

RALF1, RALF22, and RALF23 each respectively bind to FER to regulate root growth and cope with abiotic and biotic stress [9,10,11]. Haruta et al. (2014) first identified that RALF1 binds directly to FER to inhibit root elongation [9]. Stegmann et al. (2017) demonstrated that FER acts as a scaffold regulated by RLAF23 to control immune signaling [10]. Zhao et al. (2018) showed that under high salt stress, FER, together with RALF22/23, and LRX3, LRX4, and LRX5 (LRX3/4/5) leucine-rich repeat extensions, modulates cell wall integrity and ROS homeostasis, thereby enhancing salt tolerance [11]. A follow-up study by Zhao et al. (2020) further elucidated that the LRX-RALF-FER module coordinates plant growth and salt stress responses by modulating multiple hormones including ABA, ethylene, and auxin [12].

A major breakthrough came from Zhu et al. (2020), who demonstrated that FER directly interacts with the translation initiation factor eIF4E1 [13]. Upon RALF1 stimulation, FER phosphorylates eIF4E1, thereby coupling peptide signaling to spatially restricted protein synthesis- a mechanism essential for polar root hair elongation [13]. The RALF1–FER–eIF4E1 module specifically promotes the translation of mRNAs required for tip growth in root hair cells. Li et al. (2022) further revealed that RALF1 triggers biphasic root growth inhibition upstream of auxin biosynthesis, adding another layer of hormonal integration [14].

However, whether the translational control mechanism operates under abiotic stress conditions, and whether it contributes to stress tolerance beyond root hair morphogenesis, have not been investigated. Abiotic stresses such as heavy metal toxicity and high salinity are major constraints on agricultural productivity and perturb root development [15,16]. Li et al. (2022) reported that a cytoplasmic glutathione reductase BcGR1.1 enhances tolerance to copper stress in Arabidopsis [15]. Liu et al. (2024) reviewed the fact that RALF peptides play small but powerful roles in plant adaptive and developmental responses to various stresses [16]. When exposed to NaCl treatment, the growth of *ralf1* mutant plants is suppressed compared to the wild type, as noted by Chowdhary and Songachan (2025) [17]. FER, RALF22/23, and LRX3/4/5 work together in response to high salt stress [11,12]. Nevertheless, no systematic study has been conducted on how the simultaneous disruption of the *RALF1* and *eIF4E1* genes influences plant stress tolerance. Addressing this knowledge gap will provide new insights into how receptor-mediated peptide signaling cooperatively integrates abiotic stress factors into plant growth and development.

Beyond root hair development and stress responses, FER has been implicated in flowering regulation. Wang et al. (2020) showed that FER was involved in the alternative splicing of certain flowering-related genes, and that *fer* mutants exhibited delayed flowering under long-day conditions [18]. Additionally, the eukaryotic translation initiation factor eIF4E1 has been reported to affect flowering gene expression at the translational level [19]. The circadian clock regulates the flowering time through the *GI-CONSTANS* (*CO*)-*FLOWERING LOCUS T* (*FT*) photoperiodic pathway [20,21,22]. Suarez-López et al. (2001) established that the CO mediates between the circadian clock and flowering control [20]; Valverde et al. (2004) showed photoreceptor regulation of the CO protein [21]; and Bohlenius et al. (2006) demonstrated that the CO/FT module controls flowering timing in trees [22]. Meanwhile, FLC inhibits *FT* and *SUPPRESSOR OF OVEREXPRESSION OF CONSTANS 1* (*SOC1*) expression, playing a crucial inhibitory role in vernalization and autonomous pathways [18]. Whether the RALF1-FER-eIF4E1 signaling module feeds into these flowering regulatory networks remains unexplored.

In the present study, we generated *Arabidopsis ralf1*/*eif4e1* double mutants and characterized their root hair phenotypes under various adverse stress treatments. Our results further elucidate how *RALF1* and *eIF4E1* synergistically contribute to root hair formation, abiotic stress adaptation, and potentially the flowering regulation mechanism via the RALF1-FERONIA-eIF4E1 module signaling pathway.

## 2. Results

### 2.1. Identification of Homozygote ralf1/eif4e1 Double Mutants in Arabidopsis

An approximately 1.2 kb band was amplified with LP-RP primer pairs from wild-type and heterozygous lines for the *RALF1* gene using wild-type DNA as a template. In contrast, an approximate 650 bp fragment was obtained for the *RALF1* gene with RP-LBb1.3 primer pairs. The three-primer method was used to identify whether the F_1_ generation was a double-mutant heterozygote or homozygote (Figure S1).

**Figure 1:**
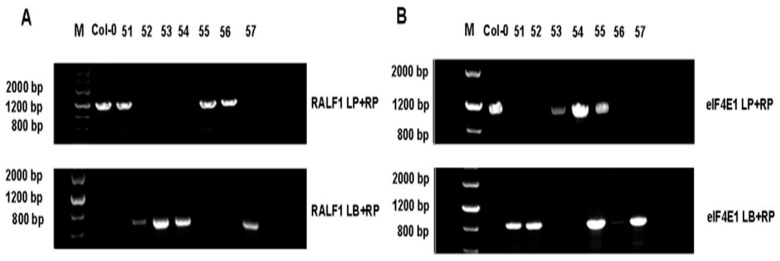
Identification of F2 *ralf1*/*eif4e1* homozygous double mutant by three-primer method. (**A**) RALF1 LP + RP reaction and RALF1 LB + RP reaction. *ralf1*/*eif4e1* F2 samples are 51–57. (**B**) eIF4E1 LP + RP reaction and eIF4E1 LB + RP reaction. Numbers #51–57 are *ralf1*/*eif4e1* F2 samples. Col-0 is wild type as control and M is marker.

Genomic DNA was extracted from individual F_2_ plants, and a three-primer method was used to identify homozygous plants. Ultimately, plants 52 and 57 were identified as homozygous for the *ralf1*/*eif4e1* double mutation (Figure 1).

**Figure 2:**
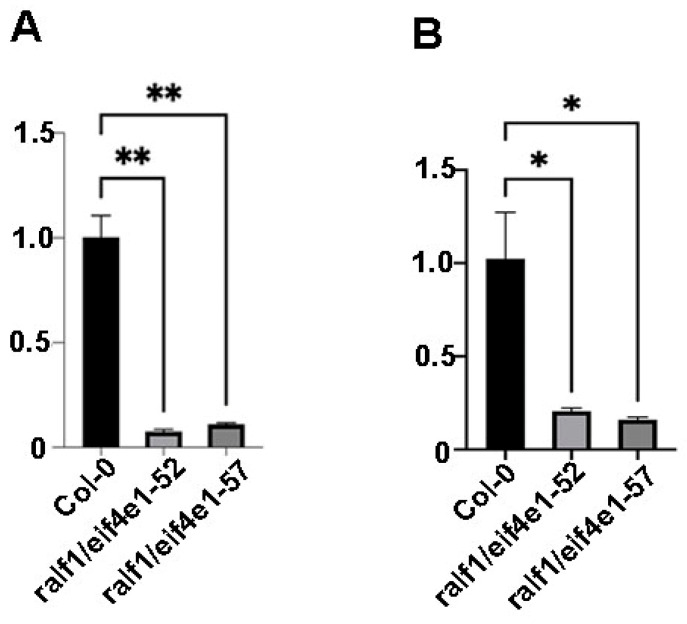
The mRNA levels of RALF1 and eIF4E1 in *ralf1*/*eif4e1* double mutants by qPCR. (**A**) With pairs of q-*ralf1* primers; (**B**) with pairs of q-*eif4e1* primers. Note: * *p* < 0.05, ** *p* < 0.01, *t*-test for samples.

Additionally, the expression levels of *RALF1* and *eIF4E1* in the F_2_ generation plants were determined. In the present study, our qPCR research results indicated that the expression levels of *RALF1* and *eIF4E1* in both the 52 and 57 double mutants were seriously decreased (Figure 2), and the double mutants had been successfully obtained.

### 2.2. Responsiveness of ralf1, eif4e1, and ralf1/eif4e1 Mutants to NaCl and ABA Treatment During Early Seedling Growth

The seed germination assays indicated that under control conditions, there were no obvious differences in the seed germination rates of each mutant compared with wild type (Col-0) (Figure S2). Similarly, there was no difference in root length among Col-0, *ralf1*, *eif4e1*, and *ralf1*/*eif4e1* lines with 100 mM NaCl treatment (Figure S3). However, after 150 mM NaCl treatment, the root length of *ralf1/eif4e1* double mutants was more sensitive to NaCl than Col-0, *ralf1*, and *eif4e1* lines (Figure S3).

**Figure 3:**
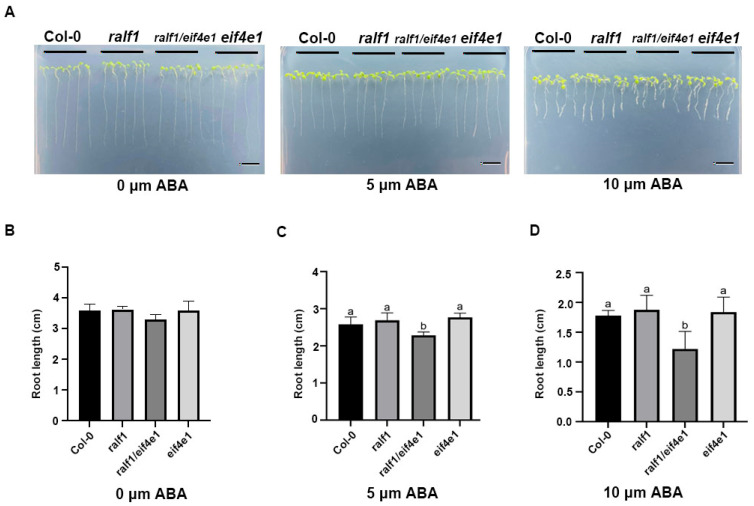
Early seedling growth of *ralf1*, *eif4e1*, and *ralf1*/*eif4e1* mutants and wild-type (Col-0) plants in response to ABA treatment. (**A**) Phenotypes of Col-0, *ralf1*, *eif4e1*, and *ralf1*/*eif4e1* seedling responses to ABA treatments (1/2 MS; 1/2 MS with 5 μm ABA; 1/2 MS with 10 μm ABA). (**B**) Root lengths of ralf1, eif4e1, and ralf1/eif4e1 mutants and Col-0 grown under normal conditions on half-strength MS medium. (**C**) Root lengths of *ralf1*, *eif4e1*, and *ralf1*/*eif4e1* mutants and Col-0 grown on half-strength MS medium with 5 μm ABA. (**D**) Root lengths of *ralf1*, *eif4e1*, and *ralf1*/*eif4e1* mutants and Col-0 grown on half-strength MS medium with 10 μm ABA. Values are means + SD of three replicates (at least 40 seeds were used for each replicate). Different letters indicate significant differences at *p* < 0.05. Scale bar = 100 μm.

We tested the roles of *RALF1* and *eIF4E1* in ABA signaling using T-DNA insertion mutants, including *ralf1*, *eif4e1*, and *ralf1*/*eif4e1*. Under ABA treatment conditions, statistical analysis revealed that only the *ralf1*/*eif4e1* double mutant had shorter root length and was sensitive to ABA treatment (Figure 3). However, whether the ABA concentration was 5 μM or 10 μM, there was no significant difference between *ralf1* and *eif4e1* in seedling growth when ABA treatment was carried out (Figure 3). These data indicated that *RALF1* and *eIF4E1* played a negative regulator role in *Arabidopsis* seedling responses to NaCl and ABA treatment.

### 2.3. eIF4E1 and RALF1 Affect Seedling Root Growth Under Cadmium Stress

**Figure 4:**
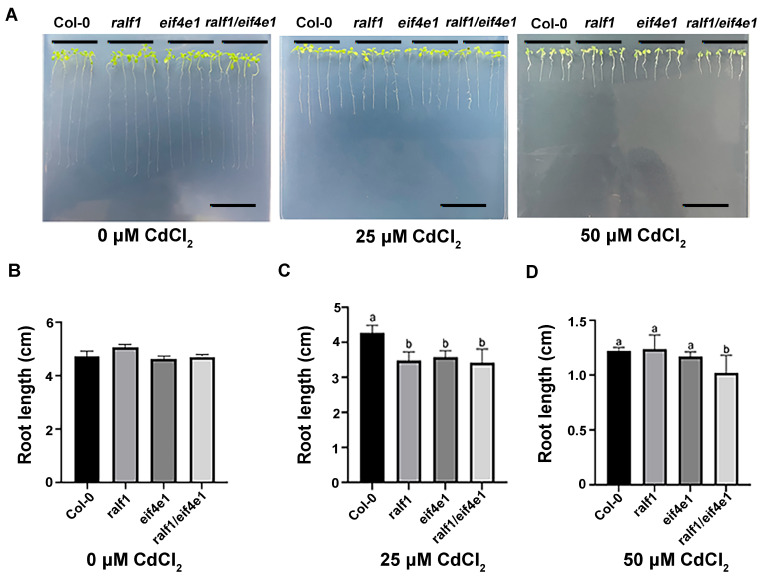
Seedling root growth of *ralf1*, *eif4e1*, and *ralf1*/*eif4e1* mutants and wild-type (Col-0) plants in response to cadmium treatment. (**A**) Phenotypes of Col-0, *ralf1*, *eif4e1*, and *ralf1*/*eif4e1* seedling root growth under cadmium treatments (1/2 MS; 1/2 MS with 25 μM CdCl_2_; 1/2 MS with 50 μM CdCl_2_). (**B**) Root lengths of *ralf1*, *eif4e1*, and *ralf1*/*eif4e1* mutants and Col-0 grown under normal conditions on half-strength MS medium. (**C**) Root lengths of *ralf1*, *eif4e1*, and *ralf1*/*eif4e1* mutants and Col-0 grown on 1/2 MS medium with 25 μM CdCl_2_. (**D**) Root lengths of *ralf1*, *eif4e1*, and *ralf1*/*eif4e1* mutants and Col-0 grown on 1/2 MS medium with 50 μM CdCl_2_. Values are means ± SD of three replicates (at least 40 seeds were used for each replicate). Different letters indicate significant differences at *p* < 0.05. Scale bar = 100 μm.

To observe the phenotypic differences in seedling root growth under cadmium stress, 7-day-old seedlings of Col-0, *ralf1*, *eif4e1*, and *ralf1*/*eif4e1* lines were treated with CdCl_2_ (0, 25, 50 μM) for 7 days. Under 25 μM Cd^2+^ treatment, the root lengths were reduced in all genotypes compared to the control (0 μM), but the reduction was statistically significant only for the *ralf1*/*eif4e1* double mutant; those of *ralf1*, *eif4e1*, and *ralf1*/*eif4e1* were 30.0%, 22.3%, and 26.3% shorter than the corresponding mutant lines (without CdCl_2_ treatment), respectively. Notably, the double mutant line of *ralf1*/*eif4e1* was sensitive to higher concentrations of Cd^2+^ at 50 μM. In particular, the root lengths were 78.0% less short than those of the untreated double mutant lines (Figure 4). On the whole, loss of function of these mutations resulted in enhanced sensitivity of root length to CdCl_2_ stress in *ralf1*, *eif4e1*, and *ralf1*/*eif4e1* mutant lines.

### 2.4. eIF4E1 Is Required for RALF1-FER Pathway-Mediated Regulation of RH Size

**Figure 5:**
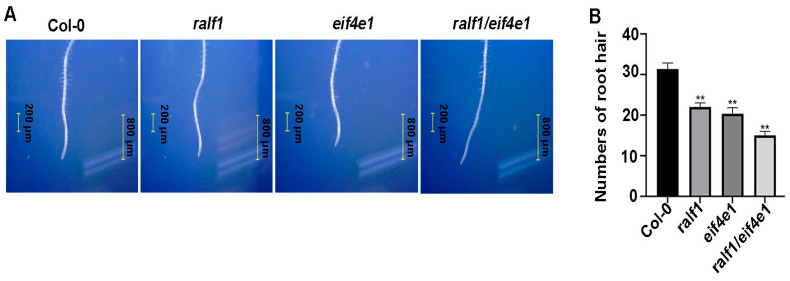
*RALF1* and *eIF4E1* genes mediate RALF-FER-eIF4E1 signaling during RH growth. (**A**) Representative images of root hair morphology from 4-day-old seedlings. (**B**) Statistics of root hair numbers in *Arabidopsis* meristematic zone. Note: ** *p* < 0.01, *t*-test for samples. Values are means ± SD of three replicates.

**Figure 6:**
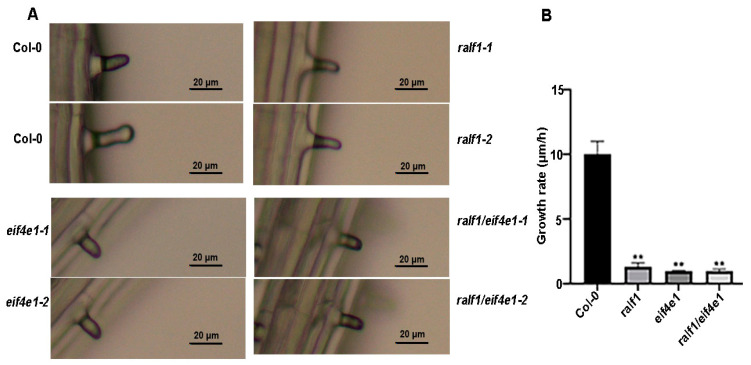
eIF4E1 functions downstream of RALF1-FER to regulate cell size. (**A**) Representative images of individual root hairs from the meristematic zone of Arabidopsis seedlings. (**B**) Average growth rate of root hairs in *Arabidopsis* meristematic zone. Note: ** *p* < 0.01, *t*-test for samples. Values are means ± SD of three replicates.

To investigate whether eIF4E proteins are necessary for the regulation of RH cell size by the RALF1-FER pathway, we examined the RH phenotypes of *ralf1*, *eif4e1* and *ralf1*/*eif4e1* mutant plants. The *ralf1*/*eif4e1* double mutant severely affected RH growth, producing a distinct phenotype with shorter and fewer root hairs compared to other mutants and wild-type plants (Figure 5A). The microscopic observation results showed that root hair numbers visually decreased significantly in root hairs for *ralf1*, *eif4e1*, and *ralf1*/*eif4e1* double mutants compared to the wild types (Figure 5B). Consistent with the previously established role of the RALF-FER-eIF4E1 axis in root hair growth under control conditions, our results from Figure 5 and Figure 6 show that losses of RALF1 and eIF4E1 jointly regulate root hair growth through the RALF-FER-eIF4E1 pathway [13].

### 2.5. eIF4E1 and RALF1 Genes Play a Key Role in Root Hair Growth

To further explore our understanding of how *eIF4E1* and *RALF1* genes modulate root hair growth, the root hairs that had just emerged from the root meristems of *Arabidopsis* were selected for measurement. That is to say, the changes in root hair length per hour were recorded to calculate their growth rate. After root tips formed bulges, WT RHs converted to tip growth, exhibiting a stable growth rate of 10.57 ± 0.29 μm/h; *ralf1* RHs grew at 1.13 ± 0.15 μm/h; *eif4e1* RHs grew at 0.81 ± 0.13 μm/h; whereas *ralf1*/*eif4e1* grew at 0.72 ± 0.21 μm/h (Figure 6). This indicates that *RALF1* and *eIF4E1* absence inhibits the growth rate of *Arabidopsis* root hairs, suggesting that *RALF1* and *eIF4E1* genes jointly regulate root hair growth through the RALF-FER-eIF4E1 pathway.

### 2.6. RALF1 and eIF4E1 Genes Affect Flowering Time

**Figure 7:**
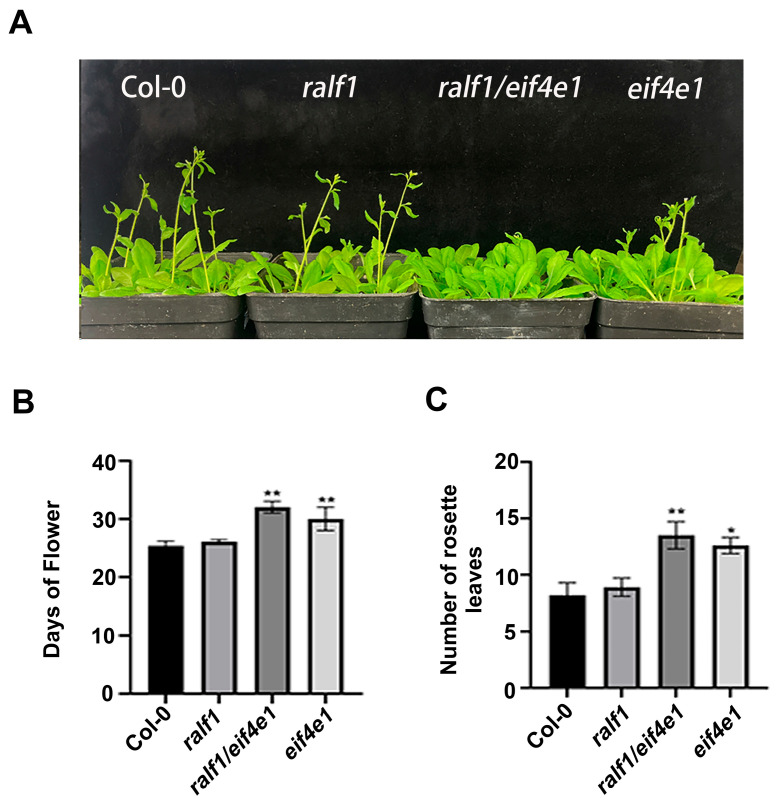
Morphology of wild type (Col-0), *ralf1*, *eif4e1*, and *ralf1*/*eif4e1* double mutant lines. Data were collected at LD conditions. (**A**) Phenotypes of wild type (Col-0), *ralf1*, *eif4e1*, and *ralf1*/*eif4e1*; (**B**) days to flowering; and (**C**) total number of rosette leaves. Note: * *p* < 0.05, ** *p* < 0.01, *t*-test for samples. Values are means ± SD of three replicates.

To explore the role of loss of function of *RALF1* and *eIF4E1*, the flowering time and numbers of rosette leaves were compared with WT and *ralf1*, *eif4e1*, and *ralf1*/*eif4e1* mutant plants. Under normal conditions, *eif4e1* and *ralf1*/*eif4e1* mutant plants showed a significant differences and flowered much later than the wild-type and *ralf1* mutant plants. At the same time, both *eif4e1* and *ralf1*/*eif4e1* mutant plants delayed flowering for several days with more rosette leaves (Figure 7). Consistent with the previous findings of Zhang et al. (2024) [19], loss of function of the eIF4E1 gene leads to delayed flowering.

**Figure 8:**
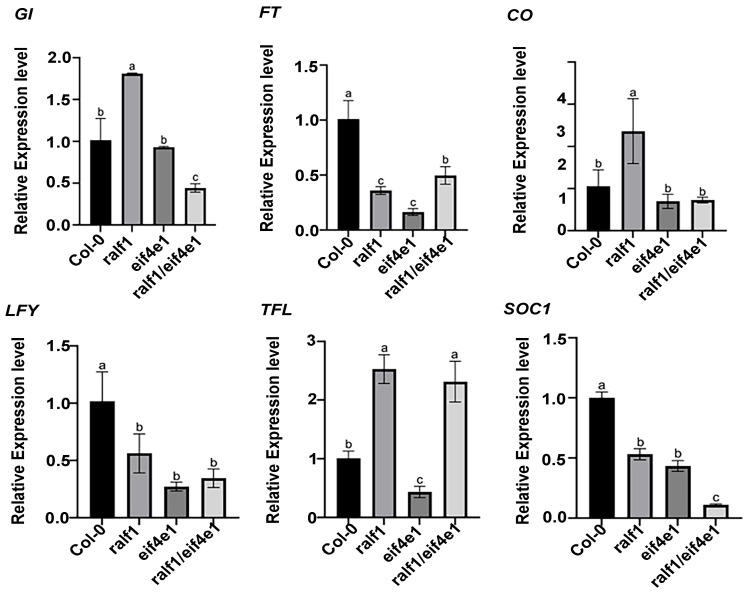
The quantitative real-time RT-PCR analysis of different flowing time genes and their expression levels in various mutant plants. The transcript level of each gene was determined using the qRT-PCR method and was then normalized with the transcript level of *ACTIN8*. Values are means ± SD of three replicates. Different letters indicate significant differences at *p* < 0.05.

In order to further investigate the impact of *RALF1* and *eIF4E1* on flowering, we determined the transcript levels of flowering-related marker genes (*SOC1*, *TFL1*, *LFY*, *GI*, *FT*, and *CO*) by qRT-PCR. The results showed that *FT*, *SOC1*, and *LFY* mRNAs were highest in wild-type plants, followed by in *ralf1* mutant plants. *GI* and *CO* were highest in *ralf1* mutants, and *TFL1* was higher in *ralf1* and *ralf1*/*eif4e1* mutants (Figure 8). These findings are consistent with the known antagonistic roles of *FT* (flowering activator) and *TFL1* (repressor).

## 3. Discussion

The RALF1-FER-eIF4E1 signaling module has been established as a key regulator of polar root hair growth through localized translational control [13,14]. However, whether this module integrates environmental stress signals and coordinates developmental transitions such as flowering has remained unexplored. Here, we generated *ralf1/eif4e1* double mutants and provided multiple lines of evidence that RALF1 and eIF4E1 synergistically regulate not only root hair elongation but also abiotic stress tolerance and flowering time. Our findings position this module as a convergence point linking extracellular peptide perception, mRNA translation, and whole-plant developmental responses to adverse environments.

### 3.1. RALF1 and eIF4E1 Cooperatively Promote Root Hair Elongation via Translational Control

Our observation that *ralf1/eif4e1* double mutants exhibit a more severe reduction in root hair density and elongation than either single mutant is consistent with the model that RALF1 and eIF4E1 act in the same pathway rather than parallel ones [13]. Zhu et al. (2020) demonstrated that FER phosphorylates eIF4E1 upon RALF1 binding, thereby enhancing the translation of tip-growth mRNAs such as *RHD6*, *ROP2*, and *RSL4* [13]. The synergistic phenotype of the double mutant supports the idea that RALF1-mediated FER activation is the primary upstream signal for eIF4E1 function in root hairs. However, the existence of RALF22, which regulates root hair growth independently of FER via pectin interactions [23], suggests that root hair development is controlled by at least two parallel modules: an FER-dependent translational module (RALF1-FER-eIF4E1) and an FER-independent cell wall integrity module (RALF22–pectin). Whether these modules converge on common downstream targets (e.g., RSL4 protein levels) remains an open question. Future ribosome profiling of *eif4e1* and *ralf1* single and double mutants would reveal the extent to which RALF1-FER signaling specifies the translatome of root hair cells.

### 3.2. The RALF1-FER-eIF4E1 Module Confers Stress-Specific Tolerance

The double mutant displayed enhanced sensitivity to Cd^2+^, ABA, and high salinity. This stress-specific pattern is informative. First, the unaltered response to Cu^2+^ indicates that the module does not globally affect heavy metal detoxification. Both Cd^2+^ and salinity induce cell wall damage and activate the LRX-RALF-FER module [11,12]. In contrast, Cu^2+^ is primarily chelated by metallothioneins and transported by COPT family proteins, pathways not obviously linked to FER [15]. Second, the enhanced ABA sensitivity in the double mutant is intriguing because FER has been reported to inhibit ABA responses via the GEF1/410-ROPP11-ABI2 signaling network [7,24]. Loss of FER leads to ABA hypersensitivity. Our *ralf1/eif4e1* double mutant phenocopies this hypersensitivity, suggesting that RALF1 and eIF4E1 are both required for FER-mediated suppression of ABA signaling. However, we did not observe a significant ABA response change in single mutants, implying functional redundancy or that the double mutant disrupts both the upstream ligand (RALF1) and the downstream effector (eIF4E1), fully abrogating the pathway. A key limitation is that we measured only whole-seedling ABA responses; tissue-specific (e.g., root tip) ABA sensitivity may differ. Future experiments should examine ABI2 phosphorylation status and ROS production in the double mutant to localize the genetic interaction.

### 3.3. Delayed Flowering in the Double Mutant Reveals a Novel Role for the RALF1-FER-eIF4E1 Module in Floral Transition

One of our most striking findings is that *ralf1/eif4e1* double mutants flower later and produce more rosette leaves than wild-type plants, with altered expression of flowering regulators. Specifically, qPCR analysis showed that *FT*, *LFY*, and *SOC1* transcripts reached maximum levels in wild-type plants, whereas expression of certain floral meristem identity genes was higher in the *ralf1* single mutant than in wild types. This complex expression pattern suggests that RALF1 and eIF4E1 influence flowering through multiple nodes. Wang et al. (2020) reported that *fer* mutants exhibit delayed flowering under long-day conditions due to altered alternative splicing of flowering-related genes [18]. Our results extend this model by implicating eIF4E1-dependent translation. Because we only measured steady-state mRNA levels, we cannot distinguish between transcriptional and translational effects. However, given that eIF4E1 is a translation initiation factor, it is plausible that RALF1-FER signaling promotes the translation of key flowering activators (e.g., *CO*, *FT*, or *SOC1*) under inductive conditions. When eIF4E1 is absent, even if *FT* mRNA is present, its protein product may be limiting. This hypothesis is consistent with the known role of eIF4E1 in selectively translating specific mRNA cohorts [13,19]. Zhang et al. (2024) showed that for *eif4e1* single mutants, both total RNA and translation efficiency analyses are required to fully understand flowering gene regulation [19]. We propose a testable model: under long-day conditions, RALF1-FER signaling activates eIF4E1, which then preferentially translates *FT* and *SOC1* mRNAs, accelerating flowering. In the double mutant, this translational boost is lost, leading to delayed flowering.

### 3.4. An Integrated Model: The RALF1-FER-eIF4E1 Module as a Stress-Responsive Developmental Switch

How might the same signaling module regulate root hair growth, stress tolerance, and flowering? We propose that the RALF1-FER-eIF4E1 axis acts as a translational checkpoint that couples environmental stress perception to developmental progression. Under favorable conditions, basal RALF1-FER signaling maintains eIF4E1 activity, supporting translation of root hair mRNAs (for nutrient uptake) and flowering activators (for timely reproduction). Under abiotic stress (high salinity, Cd^2+^, or ABA), RALF1-FER signaling is suppressed [25], reducing eIF4E1-dependent translation. This results in three coordinated outcomes: (1) inhibition of root hair elongation (conserving resources), (2) enhanced ABA sensitivity (promoting stress acclimation), and (3) delayed flowering (avoiding reproduction under unfavorable conditions). The specificity of the stress response (e.g., no effect on Cu^2+^) likely arises from the distinct cell wall perturbations triggered by different stresses, which differentially modulate FER kinase activity. Our double mutant phenocopies the “stress-always” state, showing constitutive defects in root hairs, ABA hypersensitivity, and late flowering even under control conditions.

## 4. Material and Methods

### 4.1. Plant Material and Growth Conditions

T-DNA insertion lines, *ralf1* (SALK_089792) for *RALF1* (AT1G02900) and *eif4e1* (SALK_067430C) for *eIF4E1* (AT4G18040), were obtained from Fuzhou Airosa Biotechnology Co., Ltd. (Fuzhou, China). The seeds of both the wild type (Col-0) and loss of function mutant lines were surface-sterilized with 75% ethanol (30 s), 15% (*v*/*v*) sodium hypochlorite was subsequently added for seed disinfection (12 min), and the samples were then rinsed with sterile water three times. The sterilized seeds were placed in the refrigerator and exposed to cold treatment at 4 °C for 3 days. Finally, the seeds were transferred to 1/2 Murashige Skoog (MS) medium in the sterile environment. The medium plates were then transferred to a growth chamber at 24 °C and cultivated under a light/dark (16/8 h) cycle. Eight-day-old seedlings were transplanted into a soil mixture (vermiculite: nutrient soil = 1:1) under greenhouse conditions (24 °C, 40% humidity) with a light/dark (16/8 h) cycle.

### 4.2. Obtainment of Double Mutants and Homozygous Identification

The homozygous *ralf1* (SALK_089792) and *eif4e1* (SALK_067430C) single-insertion mutants were hybridized to obtain *ralf1*/*eif4e1* double mutants [26]. The identification of homozygous lines of the T-DNA insertion mutants was based on the three-primer PCR method. With the genomic DNA of *Arabidopsis* leaves as the template, PCR amplification reaction was carried out using DNA polymerase with the designed primers. The designs of T-DNA primers were based on the left/right border sequences of the T-DNA insertion mutants. The *RALF1* gene-specific primers flanking the reported insertion sites in *ralf1* were: *RALF1* LP: 5′-AAACATGTTGCGAATTTTGC-3′; *RALF1* RP: 5′-CAATAGCAGAGTGTATCGGG-3′. The *eIF4E1* gene-specific primers flanking the reported insertion sites in *eif4e1* were: *eIF4E1*-LP: 5′-GCAGGCAGATCACAACCTTTAG-3′; *eIF4E1*-RP: 5′-AATGGGATCTTCTAATCCCC-3′. The T-DNA primer was: LBb1.3, 5′-TTCACCACTGCAACCACTAC-3′.

### 4.3. Seed Germination Assay

The surface-sterilized seeds were vernalized for 3 days prior to sowing. The plates were then incubated in the illumination incubator at 20 °C with 16/8 h (light/dark) for 7 days for germination analysis. Germination was defined as visible radicle emergence, and the cotyledon greening rate was recorded by calculating the seedlings with completely green cotyledons at certain time intervals.

### 4.4. Root Growth Analysis

After germination for the root growth assay, forty seeds of each genotype were sown in Petri dishes containing 1/2 MS medium for 7 days, and then the seedlings were transferred to new Petri dishes for vertical culture under heavy metal [16,27,28], NaCl [29], and ABA [7,30] stress, which were sealed with parafilm under strict aseptic conditions. After 7 days of adverse treatment, the root lengths of all the mutants were recorded.

### 4.5. Root Hair Phenotypic Analysis

The surface-sterilized and vernalized *Arabidopsis* seeds of *ralf1*, *eif4e1* and the *ralf1/eif4e1* double mutant were germinated on vertically oriented 1/2 MS solid agar plates. For root hair density assessment, seedlings were cultivated for 4 days under controlled conditions. The length and numbers of root hairs approximately 0.8–1.6 mm away from the root tip were determined using an Olympus SZX16 stereomicroscope (Olympus Corporation, Tokyo, Japan). High-resolution images were continuously collected for 1 h in total. The growth rate of root hairs was determined and analyzed using ImageJ-1.54p software (ImageJ, U.S. National Institutes of Health, Bethesda, MD, USA, https://imagej.net/ij/). Each variety had more than eight seedlings (*n* ≥ 8, 80–200 root hairs) and was recorded for measurement data and statistically analyzed.

### 4.6. Determination of Flowering Time

The assessment of rosette leaves was carried out by growing in the nutrient soil with an 80–100 μmol m^−2^s^−1^ light intensity at 22 °C in the greenhouse. In each individual experiment, more than 40 plants of each genotype were planted. The flowering time was determined through three biological replicates based on the visible flower buds at the center of the rosette once the bolt was 0.5 cm tall and the days from germination to flowering.

### 4.7. Real-Time Quantitative PCR

Total RNA was extracted from 2-week-old seedlings using TRIzol (Invitrogen, Waltham, MA, USA). RNA purity (A260/A280: 1.9–2.1) and integrity (agarose gel) were verified. First-strand cDNA was synthesized from 1 μg RNA using PrimeScript RT Kit (Takara, Aoto, Katsushika, Tokyo, Japan) with oligo-dT and random hexamers (37 °C, 15 min; 85 °C, 5 s). qRT-PCR was performed using TB Green Premix Ex TaqII (Takara) on a CF × 96 system (Bio-Rad, Hercules, CA, USA). Each 20 μL reaction contained 2 μL cDNA, 0.8 μM primers, and 10 μL 2× Premix. Cycling: 95 °C for 30 s; 40 cycles of 95 °C for 5 s; and 60 °C for 30 s, followed by melt curve analysis. Primers are listed in Table 1. The relative expression levels of flowering-related genes were quantified with the 2^−ΔΔCT^ method [31]. *Actin8* served as internal control.

### 4.8. Statistical Analysis

One-way ANOVA was performed on the data using IBM SPSS 26.0 software, and Student’s *t*-tests were adopted. Significant differences were defined as a *p* value less than 0.05. In the graphs of the research work, * *p* < 0.05 and ** *p* < 0.01 were marked, respectively. Duncan’s test, with a 5% probability, was utilized to distinguish differences between varieties.

## 5. Conclusions

The results underscore the RALF1-eIF4E1 signaling axis as a critical regulatory node coordinating root hair development, stress adaptation, and reproductive timing. The *ralf1*/*eif4e1* double mutant exhibits reduced root hair density and growth velocity compared to wild-type plants, implicating their cooperative roles in root development. The adversity-induced stress phenotype revealed heightened sensitivity of the double mutant to Cd^2+^, NaCl, and ABA treatments, but unchanged responses to Cu^2+^. This research offered a new avenue for improving crop stress tolerance through genetic manipulation of peptide-receptor-translational networks.

## Figures and Tables

**Table 1:** Primers for qRT-PCR of flower-related genes and identification of *ralf1*/*eif4e1* double mutation homozygotes.

Genes	Forward Primers (5′→3′)	Reverse Primers (5′→3′)
*RALF1*	ATCTCTTCACCTCCGTCCA	AGGCACACTGTTCCGTTTCA
*eIF4E1*	GAAGAAGGAGAGATGCCG	TAAACACGGGTCCAAGGA
*GI*	CAGCTGATAGACTCGCAGGG	TCGACCACTGCTAGTCCAGA
*FT*	TGTGTAGAGGGTTCATGCCT	CACCCTGGTGCATACACTGT
*CO*	TCTTACAGTTCCACGGCCAC	AGCATCTGAGCTGGAGGGA
*SOC1*	CTCTCAGTGCTTTGTGATGCT	CGATTGAGCATGTTCCTATGCC
*LFY*	ATCGCTTGTCGTCATGGCTG	GCAACCGCATTGTTCCGCTC
*TFL1*	CCAAGGCCAAGCATAGGGAT	GCGGTTTCTCTTTGTGCGTT
*Actin8*	TCAGCACTTTCCAGCAGATG	CTGTGGACAATGCCTGGAC

## Data Availability

The original contributions presented in this study are included in the article/Supplementary Material. Further inquiries can be directed to the corresponding author.

## Supplementary Materials

The following supporting information can be downloaded at https://www.mdpi.com/article/10.3390/plants15091369/s1, Figure S1: Identification of F1 generation ralf1/eif4e1 double mutant heterozygotes by tri-primer method; Figure S2: Seed germination of *ralf1*, *eif4e1*, *ralf1/eif4e1* mutants; Figure S3: Root growth of *ralf1*, *eif4e1*, *ralf1/eif4e1* mutants and wild type (Col-0) plants in response to NaCl treatment.
