# Supplementary material for: The RALF1–eIF4E1 Signaling Axis Mediates Root Hair Elongation, Flowering Time, and Stress Tolerance During Seed Germination and Early Root Growth in Arabidopsis thaliana
Source: Plants (Basel). 2026 Apr 30;15(9):1369. doi: 10.3390/plants15091369 (PMC13164641; doi:10.3390/plants15091369)

Supplementary materials

Figure S1 Identification of F_1 generation *ralf1/eif4e1* double mutant heterozygotes by tri-primer method. (A) RALF1 LP + RP reaction, RALF1 LB + RP reaction. Sample 1-3 are F_1 progeny of *ralf1/eif4e1* double mutant; (B) eIF4E1 LP + RP reaction, eIF4E1 LB + RP reaction. Sample 1-2 are *ralf1/eif4e1* F_1 progeny. Col-0 is the wild type as a control, and M represents Marker.

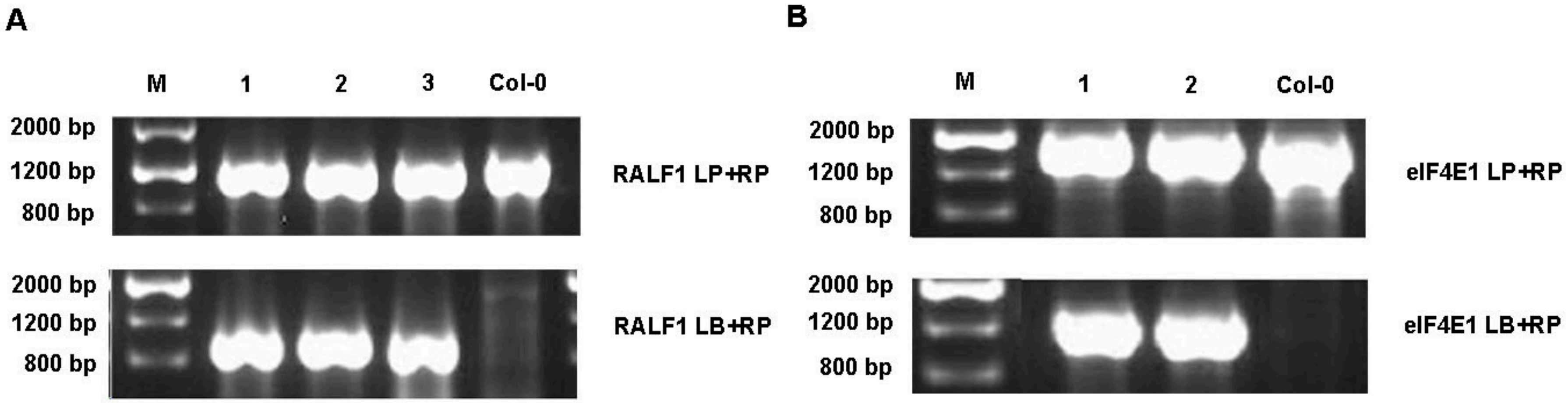

Figure S2 Seed germination of *ralf1*, *eif4e1*, *ralf1/eif4e1* mutants. (A) Phenotypes of Col-0, *ralf1*, *eif4e1*, and *ralf1/eif4e1* mutants seed germination under normal condition for 7 days. (B) Germination rates of Col-0, *ralf1*, *eif4e1*, and *ralf1/ eif4e1* mutants under normal conditions. Values are means ± SD of three replicates (at least 40 seeds were used for each replicate). Note: ns> 0.05, **P* <0.05, ***P* <0.01, t-test for samples. Scale bar=100 μm.

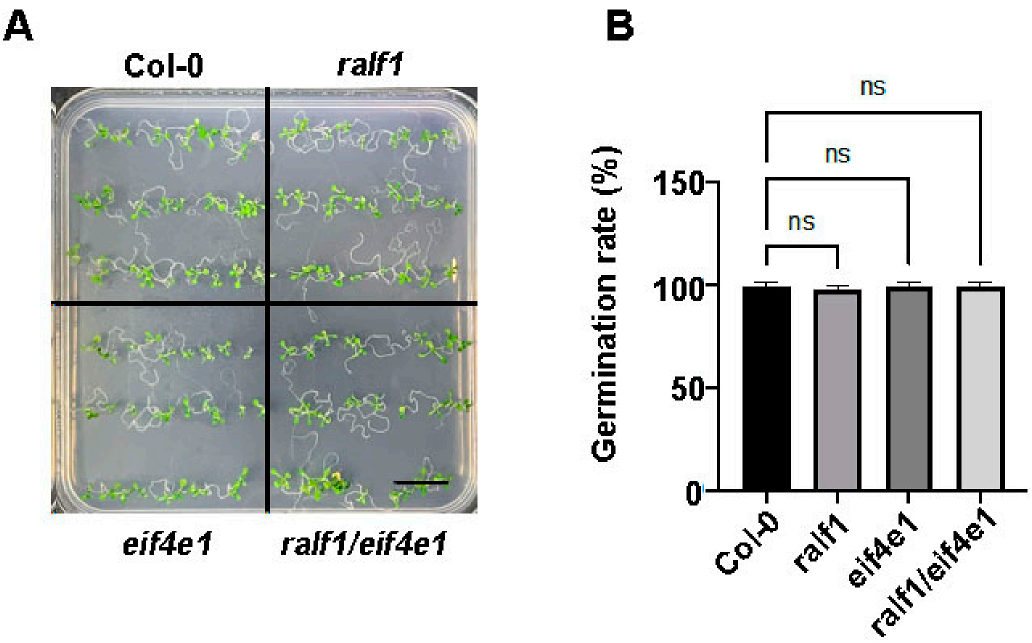

Figure S3 Root growth of *ralf1*, *eif4e1*, *ralf1/eif4e1* mutants and wild type (Col-0) plants in response to NaCl treatment. (A) Phenotypes of Col-0, *ralf1*, *eif4e1*, *ralf1/eif4e1* seed germination under NaCl treatments (1/2 MS; 1/2 MS with 100 mM NaCl; 1/2 MS with 150 mM NaCl). (B) Root length of *ralf1*, *eif4e1*, *ralf1/eif4e1* mutants and Col-0 grown under normal conditions on half strength MS medium. (C) Root length of *ralf1*, *eif4e1*, *ralf1/eif4e1* mutants and Col-0 grown on 1/2 MS medium with 100 mM NaCl. (D) Root length of *ralf1*, *eif4e1*, *ralf1/eif4e1* mutants and Col-0 grown on 1/2 MS medium with 150 mM NaCl. Values are means ± SD of three replicates (at least 40 seeds were used for each replicate). Note: ns> 0.05, **P* <0.05, t-test for samples. Scale bar=100 µm.

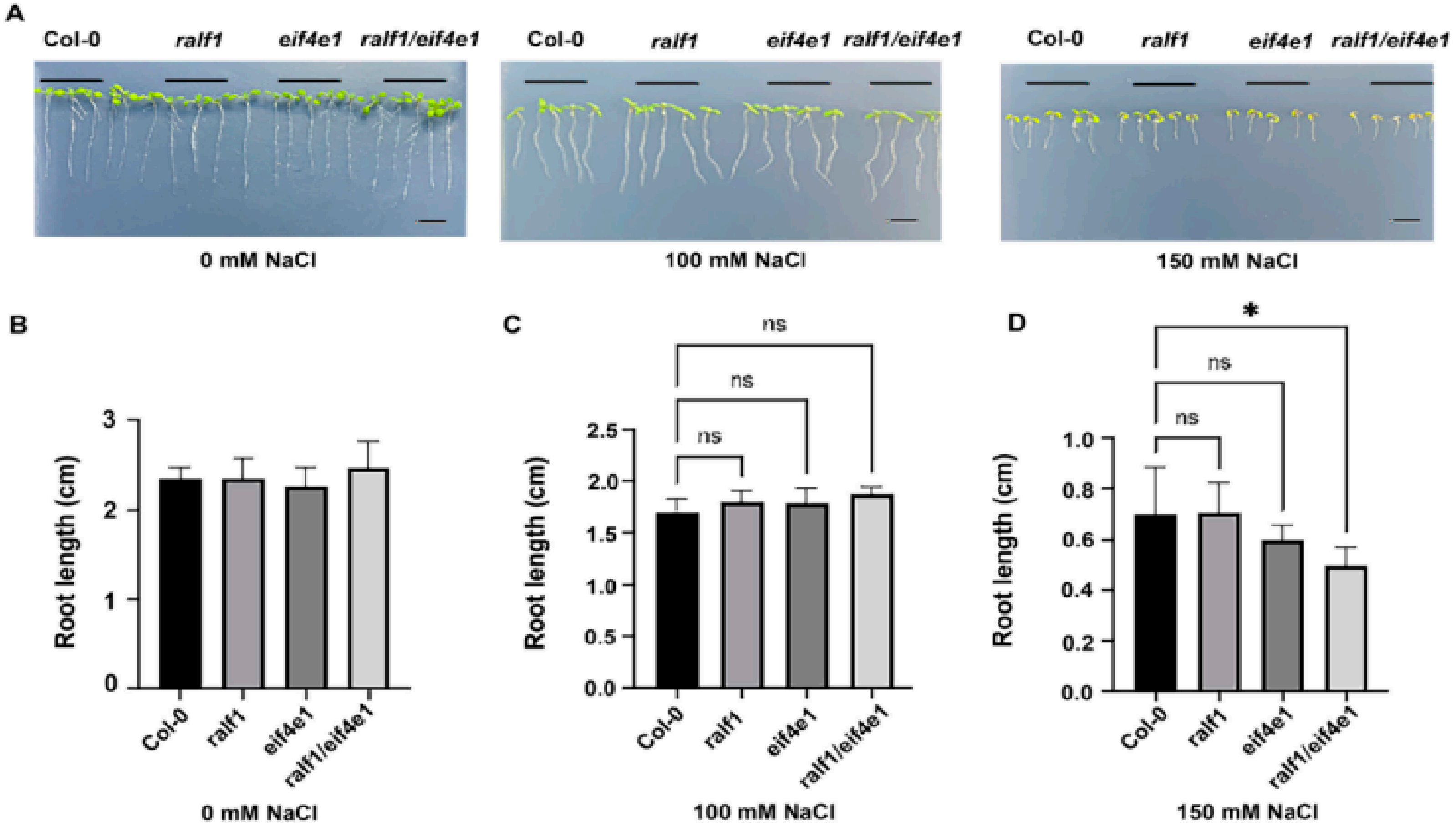

Supplement: Supplementary file 1 [file plants-15-01369-s001.zip › plants-4232124-supplementary.pdf]
